# Procoagulant Adaptation of a Blood Coagulation Prothrombinase-like Enzyme Complex in Australian Elapid Venom

**DOI:** 10.3390/toxins2061554

**Published:** 2010-06-18

**Authors:** Mettine H.A. Bos, Rodney M. Camire

**Affiliations:** 1 Department of Pediatrics, Division of Hematology, The Children’s Hospital of Philadelphia, Philadelphia, PA 19104, USA; Email: bos@email.chop.edu; 2 The University of Pennsylvania School of Medicine, Philadelphia, PA 19104, USA

**Keywords:** snake venom, blood coagulation, prothrombinase complex, factor X, factor V, prothrombin activation, serine protease, hemostatic toxin

## Abstract

The macromolecular enzyme complex prothrombinase serves an indispensable role in blood coagulation as it catalyzes the conversion of prothrombin to thrombin, a key regulatory enzyme in the formation of a blood clot. Interestingly, a virtually identical enzyme complex is found in the venom of some Australian elapid snakes, which is composed of a cofactor factor Va-component and a serine protease factor Xa-like subunit. This review will provide an overview of the identification and characterization of the venom prothrombinase complex and will discuss the rationale for its powerful procoagulant nature responsible for the potent hemostatic toxicity of the elapid venom.

## 1. Introduction

Venomous snakes produce an array of toxic compounds, including procoagulant proteins, to defend themselves and incapacitate prey. Using a murine toxicity model to compare 25 snake venoms, Broad *et al.* found in 1979 that some of the most potent venoms come from the Australian Elapidae family members *Oxyuranus microlepidotus (O. microlepidotus*; Inland taipan), *Pseudonaja textilis* (*P. textilis*; Common brown snake), and *Oxyuranus scutellatus* (*O. scutellatus*; Coastal taipan) [1].  A unique feature of their venom is that it contains substantial amounts (~5–40% of total venom protein) of procoagulant proteins with functional features similar to a macromolecular enzyme complex found in blood [2,3,4,5]. This enzyme complex, the prothrombinase complex which consists of the serine protease factor Xa (FXa) and cofactor factor Va (FVa) bound to an anionic membrane surface, plays an important role in blood coagulation as it rapidly converts prothrombin to thrombin, the latter being a key regulatory enzyme in the formation of a blood clot [6].

Prothrombin converting enzymes are commonly found in snake venoms and they are classified into four groups (A, B, C, and D) depending on their cofactor requirements in prothrombin activation [7,8,9]. Group A and B prothrombin activators are metalloproteases that convert prothrombin to the active intermediate meizothrombin; they are found in the venom of several vipers [9]. Group C and D prothrombin activators are serine proteases that are capable of fully activating prothrombin to thrombin and are exclusively found in Australian snakes [8,9]. Whereas group D activators require calcium, phospholipids, and the protein cofactor factor V (FV) for optimal protease activity, group C activators function in the absence of this cofactor. It is these group C activators that have, so far, only been observed in the Elapidae family members and have been the subject of detailed studies. These studies have shown that, remarkably, the group C activator complex consists of a FVa-like subunit and a  FXa-like subunit similar to the blood coagulation prothrombinase complex [2,4]. To date, this subset of snakes are the only species identified that have two sources of prothrombinase: one that circulates in plasma and is required for normal hemostasis and one that is present in the venom and likely plays an important role in the envenomation of prey.

This review will focus on the identification and characterization of the venom prothrombinase complex components and will discuss the biochemical rationale for its powerful procoagulant nature that is responsible for the potent hemostatic toxicity of the elapid venom.

## 2. Identification of the Venom Prothrombinase-like Complex

In 1969, it was first reported that the venoms of the Australian elapids *O. scutellatus* and *P. textilis* contain a prothrombin activator which does not require blood coagulation FV as a cofactor for efficient clotting activity [10]. This prothrombin activating complex was initially purified from *O. scutellatus* venom, and it was shown to convert prothrombin to thrombin in a manner similar to the mammalian prothrombinase complex, independent of the addition of FV [11]. A subsequent study revealed that the purified prothrombin activator is composed of two large subunits [12], which was confirmed by Speijer *et al.* whose data suggested that the multimeric complex in fact consists of a blood coagulation FXa-like catalytic subunit and a FVa-like cofactor subunit [2]. Given the comparable characteristics of prothrombin conversion for the venoms of *O. scutellatus*, *O. microlepidotus*, and *P. textilis*, it was suggested that each of these prothrombin activators have a similar multisubunit composition [2,13]. These prothrombin activator complexes from *O. scutellatus* and *P. textilis* were termed oscutarin C and pseutarin C, respectively [9].

A detailed analysis of the identity of the protein subunits came from a study by Rao and Kini, in which peptide fragments of pseutarin C were purified by HPLC and subsequently subjected to *N*-terminal sequencing [4]. Their findings confirmed that the catalytic subunit shares a high sequence homology (~60%) with mammalian FXa, whereas the cofactor subunit is highly homologous to the mammalian cofactor FVa (~55%) [4]. Subsequent preparation and sequencing of venom gland cDNA revealed that these venom proteins are encoded by two individual genes [14,15,16,17,18], which are specifically expressed in the venom gland [17,19]. As such, these members of the Elapidae snake family are unique in that they have two gene sets encoding two parallel prothrombin activating systems.

## 3. Structural Characteristics of the Venom FXa-like Catalytic Subunit

### 3.1. Blood Coagulation Factor Xa

Blood coagulation FXa is synthesized in the liver and circulates in blood as the inactive zymogen factor X (FX). Factor X consists of two chains which are held together by a disulfide bond. The  amino-terminal light chain consists of a γ-carboxyglutamic acid domain (Gla) domain and two epidermal growth factor (EGF) homology domains, and the carboxy-terminal heavy chain contains the serine protease or catalytic domain. All eleven Glu residues present in the Gla domain are posttranslationally γ-carboxylated to Gla in a process that requires vitamin K [20]. The Gla residues interact with Ca^2+^ ions and induce a conformational change that enables the protein to bind to negatively charged phospholipid membranes [21], which serves to localize the protease to a physiological cell surface [6]. The two EGF domains are involved in protein-protein interactions, while the serine protease domain comprises the active site with the catalytic triad His^57^ (the chymotrypsin numbering system is used throughout for the FXa residue numbering, unless otherwise noted [22]), Asp^102^, and Ser^195^, and several other regions involved in substrate binding and specificity, cofactor interaction, as well as Ca^2+^ and Na^+^ ion binding.

During the initiation of blood coagulation, FX is proteolytically activated by either the factor VIIIa (FVIIIa)-factor IXa (FIXa) (intrinsic tenase) or the factor VIIa (FVIIa)-tissue factor (extrinsic tenase) enzyme complexes following cleavage between Arg^15^–Ile^16^ in the heavy chain [6]. This results in the release of a large activation peptide and insertion of the newly formed heavy chain *N-*terminus Ile^16^ into the interior of the protease domain where it forms a salt bridge with Asp^194^. The subsequent conformational changes in specific functional regions of the protease domain are essential to protease maturation, resulting in a conformation optimal for substrate recognition and cofactor binding [23]. Following activation, FXa reversibly associates with its cofactor FVa on an anionic membrane surface in the presence of Ca^2+^ ions to form prothrombinase, the physiological activator of prothrombin [6].

### 3.2. The Venom-Derived Factor Xa-like Catalytic Subunit

**Figure 1:**
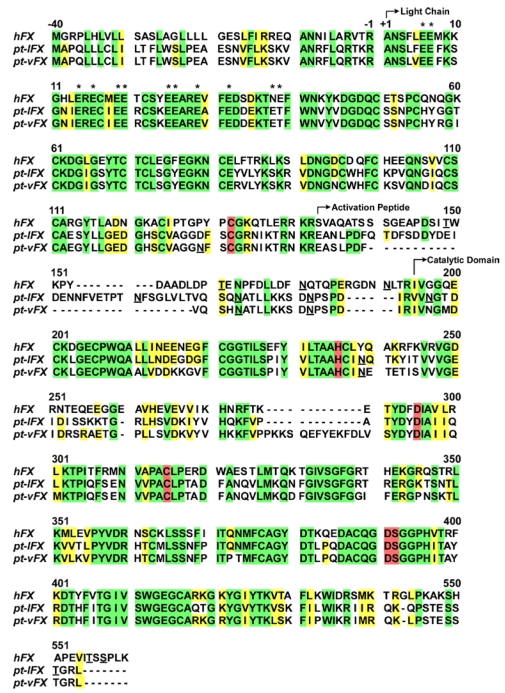
Alignment of the amino acid sequences from human FX (h-FX), *P. textilis* liver-expressed FX (pt-lFX), and *P. textilis* venom gland-expressed FX (pt-vFX) (AlignX Module; Invitrogen Carlsbad, CA, USA). Residues fully conserved between all three FX derivatives are shown in green; amino acids partially conserved between the three variants but maintained in most vertebrates are indicated in yellow. Shown in red are: the cysteines connecting the heavy and the light chains, the catalytic triad His^57^, Asp^102^, and Ser^195^, as well as Asp^194^. The start of the light chain, activation peptide, and serine protease or catalytic domain is indicated. Putative glycosylation sites are underlined, and γ-carboxylated Glu residues are indicated by * (the last Gla residue does not align between human and snake FX). The signal peptide is numbered from -40 to -1 and the mature sequence starts at +1 (regular numbering of venom FX).

Two groups have published the cDNA sequences of the FXa-like subunits expressed in the venom glands of *P. textilis*, *O. scutellatus*, and *O. microlepidotus* [15,17,18]. These venom FX sequences are very similar (91–95% sequence identity) and also share high homology with the group D prothrombin activators found in other Australian snakes (81–85% sequence identity) [17,23]; by comparison, the overall sequence identity with human FX is 49%. An alignment of the amino acid sequences of human FX, venom *P. textilis* FX, and liver-expressed *P. textilis* FX is given in Figure 1. In the next few sections, some of the structural properties of the FXa-like venom component will be discussed in  more detail.

#### 3.2.1. The Activation Peptide of Venom FX

The most striking difference between the sequences of the Australian elapid venom FXa-like proteins and blood coagulation FX is the length of the activation peptide. Even though the length of the activation peptide varies throughout evolution from 43 residues in the rat to 52 in human and 65 in zebrafish, the activation peptide of snake venom FX is considerably shorter, being 27 residues [15,17,18]. This is also considerably shorter than the FX expressed in the liver of *P. textilis*, which has an activation peptide of 56 residues (Figure 1) [24]. It is unclear if the FXa-like venom component comprises an intact activation peptide, as it was not detected upon *N-*terminal sequencing [4]. This would suggest that the activation peptide is either removed upon intracellular proteolysis of FX, or following its secretion into the venom. There is some evidence that glycosylation of the human FX activation peptide may contribute to substrate recognition by the intrinsic or extrinsic FX activating complex [25,26]. Although the activation peptide comprises several putative *N-*glycosylation sites (Figure 1), whether or not it plays a role in the proteolytic activation of venom FX similar to mammalian FX remains to be determined. 

#### 3.2.2. The Light Chain of Venom FXa

The light chain of the venom FXa-like component is very similar to that of blood coagulation FX. The Gla domain of venom FXa comprises eleven conserved Glu residues, which have been shown by chemical Gla analysis to be fully γ-carboxylated in *P. textilis* FXa (Figure 1) [27]. Furthermore, the structural elements that mediate anionic phospholipid binding are also present in the FXa-like subunit [21], which implies that, similar to mammalian FXa, venom FXa is capable of membrane binding. The structure of the EGF domains seems to be maintained as well, given that the six cysteines that form disulfide bridges characterizing the individual EGF domains are conserved. In addition, the cysteines that connect the heavy and light chains are also present in venom FXa (Figure 1).

#### 3.2.3. The Heavy Chain of Venom FXa

Other important regions of FXa that are well conserved in the snake venom FXa-like subunit include the *N-*terminal heavy chain sequence Ile^16^–Val^17^, which is essential to protease maturation [23,28], and the catalytic triad His^57^, Asp^102^, and Ser^195^ (Figure 1). Some of the regions that undergo a conformational change upon activation are highly conserved in venom FXa, such as active site loop Ala^183^–Asp^194^, which includes Asp^194^ that forms a salt bridge with Ile^16^, and the Na^+^ binding site Glu^216^–Glu^226^. In contrast, most of the residues in the catalytic domain important for Ca^2+^ coordination are lost. More intriguingly, the Ca^2+^-interactive region is followed by a 13 residue insert, Pro^251^–Leu^263^ (regular numbering of venom FX), that has been found in the venom FXa of all three elapids and, to some extent, in the group D prothrombin activators [17,24]. However, this sequence is not present in liver-expressed *P. textilis* FX, nor in other serine proteases involved in blood coagulation, such as FVIIa, FIXa, or activated protein C (APC). The functional role of this insertion is unclear.

## 4. Structural Characteristics of the Venom FVa-like Cofactor Subunit

### 4.1. Blood Coagulation Factor V

**Figure 2:**
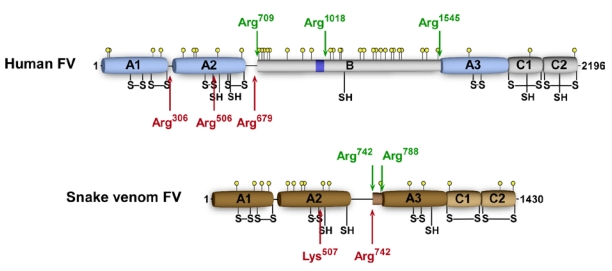
Schematic A1-A2-B-A3-C1-C2 domain representation of human and snake venom (*P. textilis*) FV. Thrombin cleavage sites are indicated by green arrows and APC cleavage sites by red arrows. Yellow circles represent potential *N-*linked glycosylation sites and the dark blue box corresponds to the basic sequence 963–1008 implicated in preserving the FV procofactor state. Disulfide bonds are indicated by S-S and free cysteines by SH.

Blood coagulation FV is a large (330 kD), heavily glycosylated, single chain protein that circulates in blood as an inactive procofactor [29]. It has an A1-A2-B-A3-C1-C2 domain structure (Figure 2), and only following removal of the large, central B domain can FV assemble or function in the prothrombinase complex [29,30]. Thrombin is considered the key physiological activator of FV and cleaves the three peptide bonds Arg^709^, Arg^1018^, and Arg^1545^ in the B domain, thereby facilitating B domain removal. The resulting cofactor, FVa, is a heterodimer composed of a heavy chain (105 kD) and a light chain (71/74 kD) that are associated through Ca^2+^ ions. Assembly of FVa with FXa in the prothrombinase complex on the cellular surface at the site of vascular injury is an essential step during blood coagulation as it dramatically enhances the rate of thrombin generation by FXa [6]. Factor Va is inactivated by APC, which cleaves at Arg^306^, Arg^506^, and Arg^679^, resulting in A2 domain dissociation and loss of FXa binding and cofactor function [29].

### 4.2. The Factor Va-like Cofactor Subunit

Similar to venom FXa, the available cDNA sequences make clear that the FVa-like cofactor subunits expressed in the venom of *P. textilis*, *O. scutellatus*, and *O. microlepidotus* are very similar (97% sequence identity) [14,16,17], whereas the sequence identity with human FV is 54%. The deduced protein sequences indicate that some of the structural features involved in FV function appear to differ significantly; these elements will be discussed in more detail in the following sections.

#### 4.2.1. The B Domain of Venom FV

A remarkable feature of the FV homologs expressed in the elapid venom is that their B domain is extraordinarily short: 46 *versus* ~600–800 residues in mammals (the FV B domain is defined as the region removed following thrombin cleavage to generate the heavy and light chains; Figure 2) [30]. Our laboratory has previously shown that the B domain plays an important function by interfering with functional binding interactions of FV through imposing steric and/or conformational constraints, thereby preventing constitutive cofactor function [31,32]. In addition, we have identified a discrete region of the B domain that plays a critical role in stabilizing the procofactor state [32]. Part of this region (residues 963–1008 of human FV) is unusually basic with 18 of 46 residues being Arg or Lys, and is well conserved across the vertebrate lineage [30]. Given that venom FV lacks the majority of the B domain sequence, it is not surprising that the so called basic region is absent as well. This intriguing observation prompted us to assess the cofactor capacity of purified recombinant venom-derived *P. textilis* FV (pt-FV) [27]. Consistent with our previous observations, we were able to show that the absence of the basic region correlates with the expression of procoagulant FV activity, indicating that venom FV is expressed as a constitutionally active FV variant [27]. As such, this is the first FV species observed thus far that exists as a constitutively active cofactor. Interestingly, liver-expressed *P. textilis* FV has a similar short B domain that lacks the basic region [19], which would suggest that the FV circulating in the elapid’s plasma is constitutionally active as well; however, biochemical studies are needed to confirm this possibility.

#### 4.2.2. The Disulfide Bond Connecting the Heavy and Light Chains of Venom FV

Sequence alignment reveals that in comparison to human FV, venom FV has lost six cysteine residues: three in the A2 domain (Cys^575^–Cys^656^, Cys^585^; human residue numbering), one in the B domain (Cys^1085^), and two free cysteines in the C1-C2 domains, of which Cys^1960^ is nonconserved throughout vertebrate evolution, whereas Cys^2113^ is only present in mammals. Venom FV has two newly introduced cysteines that are unique to Australian elapids: Cys^642^ in A2 and Cys^1002^ in A3 (snake residue numbering; Figure 2). Recent data from our laboratory show that either Cys^540^ or Cys^642^ in the A2 domain is involved in a disulfide bond that connects the heavy and light chains of pt-FV [27]. This is consistent with previous observations made by Speijer *et al.*, who obtained evidence suggesting the existence of such a linker between the heavy and light chains of *O. scutellatus* FV [2]. This disulfide bond is a unique feature of snake venom FV, since it has not been observed in any other FV species to date. Based on homology with the cysteines in human FV, Cys^1002^ could be the only free cysteine in the venom FV light chain; therefore, this residue may be part of this unique disulfide bond linking the heavy and light chains. This assumption would also imply that plasma *P. textilis* FV cannot form a similar disulfide bond, as it lacks Cys^1002^ [19].

#### 4.2.3. Activated Protein C Cleavage Sites in Venom FV

We have recently shown that *P. textilis* venom-derived FV is functionally resistant to inactivation by APC, as no loss of cofactor activity was observed upon incubation with very high concentrations of APC [27]; a similar observation was made using the venom FV-FXa complex, pseutarin C [14]. However, while the FVa functional activity was unaffected following APC treatment, we did observe proteolytic degradation of pt-FV, indicative of cleavage by APC [27]. *N-*terminal sequencing of the degradation products indicated that human APC cleaves pt-FV at Lys^507^ and Arg^742^. These sites are homologous to the APC cleavage site Arg^506^ in human FV and to Arg^709^, the thrombin cleavage site. Furthermore, our data suggested that APC cleaves pt-FV at some additional sites in the A2 region somewhere within Glu^508^–Arg^742^; however, we were unable to make the exact determination. Both the venom FV from *O. scutellatus* and *O. microlepidotus* as well as the FV expressed in the liver of *P. textilis* have similar residues at these sites, suggesting that they would be cleaved by APC in a comparable manner.

#### 4.2.4. Interdomain Connections in Venom FV

The mammalian FVa structure is stabilized by several interdomain contacts linking the C1-C2, A3-C1, and A3-C2 domains [33]. Metal ion binding further contributes to these interdomain interactions by providing stability to the local structure [33]. Analysis of the A3-C1-C2 residues involved in direct interdomain contacts indicates that all of the residues connecting the C1-C2 and A3-C2 domains are conserved in venom FV, while the hydrophobic interactions linking the A3-C1 domains are at least partially preserved. Of the residues implied in Cu^2+^ binding, the majority are found in venom FV, as well as several residues involved in essential interactions between A1 and A3. Furthermore, the high affinity Ca^2+^ binding site is completely conserved. This implies that heavy and light chains of venom FV are linked in a manner similar to mammalian FV.

#### 4.2.5. Posttranslational Modifications of Venom FV

Factor V undergoes multiple posttranslational modifications, including *N-*glycosylation, phosphorylation, and sulfation at multiple sites [29], which are not fully conserved in venom FV. Mammalian FV is heavily *N-*glycosylated, and 25 out of the 37 putative *N-*glycosylation sites are located within the B domain (Figure 1). Venom FV, on the other hand, has 16 potential *N-*glycosylation sites of which one is in the B domain (Figure 1) [34]. Some of these *N-*glysocylation sites are similar to human FV, whereas others are unique to snake FV. Interestingly, liver-expressed *P. textilis* FV has an additional putative *N-*glycosylation site in the B domain at Asn^573^ [19], which is not preserved in any of the venom FV species. Phosphorylation of the human FVa heavy chain at Ser^692^ has been implied to enhance the rate of APC-dependent inactivation [35]. Although this phosphorylation site is absent in the venom FVa-like subunit, venom-derived *P. textilis* FV could be proteolyzed by human APC as discussed above [27]. This suggests that phosphorylation of the FVa heavy chain may not be critical to inactivation by APC *per se*. Sulfation of FV has been speculated to be of importance for the recognition of FV by thrombin and for full FVa cofactor activity [36]. However, out of the six sulfation sites present in human FV, the one homologous to human Tyr^1593^ is conserved in snake venom FV. Furthermore, our data on the thrombin-mediated pt-FV activation indicate a minor role for sulfation in the recognition of venom FV by thrombin [27]. 

## 5. Evolutionary Adaptation of Prothrombinase to a Powerful Hemostatic Toxin

One of the major clinical effects of envenomation by the Australian Elapidae genera *Pseudonaja* and *Oxyuranus* is significant consumptive coagulopathy, causing early hypotension, spontaneous bleeding, and severe fibrinogen depletion [37,38,39,40]. It is thought that the injected prothrombin activating complex is responsible for these symptoms, as it rapidly converts the prey’s prothrombin to thrombin, which subsequently leads to the formation of microthrombi. This process can be life threatening in several ways: the microthrombi could get lodged in the microvasculature of the lungs, thereby resulting in a pulmonary embolism, and the formation of microthrombi severely exhausts the blood coagulation system (coagulopathy), giving rise to fibrinogen depletion and spontaneous bleedings.

This powerful procoagulant effect of the venom prothrombinase-like complex cannot be attributed to an enhanced intrinsic prothrombin converting capacity, as we have recently shown that the kinetic parameters for the conversion of human prothrombin are similar between venom-derived *P. textilis* prothrombinase and human prothrombinase [27]. Based on this, it seems likely that a combination of modifications in the protein structure as well as modulations at the level of gene transcription and subsequent protein expression have transformed the blood coagulation prothrombinase complex into a venom toxin. Several of these adaptations will be discussed in the following sections.

### 5.1. Transcriptional Regulation and Protein Expression of the Venom Prothrombinase-like Complex

Recently, Kini and coworkers have investigated the transcriptional regulation and protein expression of venom FVa and FXa. They were able to demonstrate that the expression of venom *P. textilis* FV and FX is almost 300-fold and 80-fold higher, respectively, as compared to their liver counterparts [19]. Further gene analysis suggested that this increase in expression is due to a so-called ~270 bp VERSE (Venom Recruitment/Switch Element) region in the promoter, which is thus far only found in the genes encoding venom FX of *P. textilis* and *T. carinatus* [41,42]. This indicates that the recruitment of FX to the venom gland is accompanied by one or more specific gene modulations, resulting in elevated levels of protein expression. No evidence has yet been obtained in support of a similar modification of the venom gland FV gene.

High expression of the venom prothrombinase-like components largely contributes to the hemostatic toxicity of the venom, as it allows for the rapid generation of extremely high levels of thrombin upon envenomation. The amount of venom produced by a snake when it bites (e.g., venom yield) has been found to vary from 8 to 146 mg for the *Pseudonaja* and *Oxyuranus* snakes [43]. Considering that the prothrombinase-like complex makes up 5–40% of the yield [2,3,4,5], 0.4–58 mg or 2–260 nmols (FXa-FVa ~220 kD) of the enzyme complex could be injected into the prey. Assuming that the kinetic parameters for mammalian prothrombin conversion are similar to that of human prothrombin, the amount of thrombin generated in 30 minutes can go from 20 µmols up to 2 mmols (*k_cat_* ~ 300 min^−1^) [27]. This exceeds the amount of thrombin required for clot formation at the site of injury by several orders of magnitude, which is generally assumed to be in the nanomolar range [44]. Based on these values, it is not surprising that the first symptoms of consumptive coagulopathy can be observed within half an hour following envenomation [38,40].

### 5.2. Gain-of-Function Adaptations in the *P. textilis* Venom-Derived FVa-like Subunit

We have previously found that venom FV from *P. textilis* circumvents the paradigms of normal hemostasis through a variety of changes transforming it into a potent procoagulant [27]. One of these gain-of-function elements is that pt-FV is synthesized in an active state and does not require proteolytic removal of the B domain to express procoagulant activity. As such, pt-FV is a naturally occurring example of a protein that has acquired a new functional state through loss of inhibitory sequences [30].

Another remarkable feature of pt-FV is that it binds *P. textilis* venom FXa (pt-FXa) with high affinity both in the presence and absence of anionic membranes [27]. Once assembled in solution, the FVa-FXa complex functions equivalently to the membrane-bound complex [27]. Thus, pt-FV has bypassed the restraints observed for the mammalian prothrombinase complex, which, at physiological concentrations, only assembles on a phospholipid surface such as that of platelets or damaged vascular cells. The exact mechanism underlying this requirement is generally considered to stem from a limitation in reaction dimensions due to assembly on the membrane surface [6,45]. Recently, however, it was proposed that a conformational change in the FV molecule following phospholipid binding enhances the interaction with FXa [46,47]. Our findings with the soluble *P. textilis* FVa-FXa complex would seem to support this conformational model. In the case of the venom-derived proteins, these conformational transitions are probably induced through a variety of changes to their primary structure, which mimics the structural configuration of membrane-bound FVa-FXa.

As a further procoagulant enhancement, pt-FV is functionally resistant to APC despite  APC-dependent cleavage in the heavy chain at Lys^507^ and Arg^742^ [27]. We speculate that the disulfide bond between the A2 and A3 domains contributes to stabilizing pt-FV and prevents dissociation of the A2 domain from the rest of the molecule [27]. This unique disulfide bond that connects the heavy and light chains of pt-FV may result from a newly introduced cysteine. Interestingly, although quite rare, some of the phospholipase A_2_ enzyme variants expressed in the venom of *P. textilis* and *O. scutellatus* have also been observed to have additional, nonconserved cysteines that are presumed to form intramolecular disulfide bonds [48,49]. Whether or not this unique structural feature is limited to the venom toxins of the Australian elapids *P. textilis* and *O. scutellatus* remains to be determined.

## 6. Final Remarks

Recent studies from several laboratories indicate that the prothrombinase-like complex found in the venom of a specific subset of Australian elapids has undergone several regulatory and structural modifications that account for its potent hemostatic toxicity. Uncoupling the preservation of the procofactor state of the cofactor component (FV) and bypassing the need for membrane binding to function for the *P. textilis* FVa-FXa complex are central underlying modifications allowing for the enzyme complex to rapidly activate prothrombin in solution and contribute to disseminated clotting.

The similarities between venom and plasma FV present in these snakes raise some interesting questions concerning the constitutive activity of plasma FV. Based on our data, we speculate that the liver-expressed *P. textilis* FV is already active in the absence of proteolytic activation by thrombin [27]. The implications for normal hemostasis in the snake are unclear at this point, but considering FV can only function in the presence of the active serine protease FXa, we would anticipate that the presence of an active cofactor contributes minimally to unregulated clotting.

Another interesting aspect of the presence of two almost identical enzyme systems with different functions is that the venom prothrombinase-like complex can induce coagulation in the snake’s own plasma, as has been shown previously [50]. In some cases, it has been acknowledged that snake plasma contains inhibitors that neutralize venom toxins, including those toxins affecting blood coagulation [51]. Another mechanism that protects the snake against its own venom toxins is through structural modulation of the target molecule. One of these examples can be found in the Egyptian cobra (*N. haje*) that has a unique *N-*glycosylation site in the ligand binding domain of the nicotinic acetylcholine receptor, which has been demonstrated to obstruct binding by a venom neurotoxin, but allows binding by its natural ligand [52]. We can only speculate as to whether elapid prothrombin has undergone a similar structural modification, since its sequence is not yet available.

Taken together, the prothrombinase complex represents an exceptional example of an enzyme system that has evolved into a potent biological weapon for host defense and envenomation of prey.
